# Molecular Biology of Brain Metastasis

**DOI:** 10.3390/ijms15069519

**Published:** 2014-05-28

**Authors:** Konstantina A. Svokos, Bodour Salhia, Steven A. Toms

**Affiliations:** 1 Department of Neurosurgery, Pennsylvania College of Osteopathic Medicine, 4170 City Ave. Office of Graduate Medical Education, Philadelphia, PA 19131, USA; E-Mail: konstantina.svokos@gmail.com; 2 Translational Genomics Research Institute, 445 North Fifth Street, Phoenix, AZ 85004, USA; E-Mail: bsalhia@tgen.org; 3 Department of Neurosurgery, Geisinger Health System, 100 North Academy Ave., Danville, PA 17822, USA

**Keywords:** epigenetics, tumor immunology, animal models, signal transduction

## Abstract

Metastasis to the central nervous system (CNS) remains a major cause of morbidity and mortality in patients with systemic cancer. As the length of survival in patients with systemic cancer improves, thanks to multimodality therapies, focusing on metastases to the CNS becomes of paramount importance. Unique interactions between the brain’s micro-environment, blood-brain barrier, and tumor cells are hypothesized to promote distinct molecular features in CNS metastases that may require tailored therapeutic approaches. This review will focus on the pathophysiology, epigenetics, and immunobiology of brain metastases in order to understand the metastatic cascade. Cancer cells escape the primary tumor, intravasate into blood vessels, survive the hematogenous dissemination to the CNS, arrest in brain capillaries, extravasate, proliferate, and develop angiogenic abilities to establish metastases. Molecular biology, genetics, and epigenetics are rapidly expanding, enabling us to advance our knowledge of the underlying mechanisms involved. Research approaches using cell lines that preferentially metastasize *in vivo* to the brain and *in vitro* tissue-based studies unfold new molecular leads into the disease. It is important to identify and understand the molecular pathways of the metastatic cascade in order to target the investigation and development of more effective therapies and research directions.

## 1. Introduction

Metastatic brain tumors (MBT) arise from cancers outside the central nervous system (CNS). They may reach the brain either from hematogenous spread or via direct invasion from adjacent tissues. Brain metastases are the most common brain tumors and occur nearly 200,000 times each year in the USA. They comprise about 25% of all cancer metastases and may be seen in about 20%–40% of all adult cancer patients. The number of cases for the site of origin is 40%–50% for lung, 15%–20% for breast, 5%–10% for skin, and 4%–6% for the gastrointestinal tract [1]. Brain metastases are the leading cause of morbidity and mortality in cancer patients. Median survival time for untreated patients is 5 weeks; whereas, multimodality therapy may extend survival to 3–18 months [2]. Recently, an increased incidence of MBTs has been noted. This may be a consequence of either improved imaging modalities or more effective treatment of primary tumors and longer patient survival [3].

The vast majority of MBTs occur in the cerebral hemispheres (80%) with a much smaller minority appearing in the cerebellum (15%) and the brainstem (5%). These percentages correspond to the tissue volumes of the respective portions of the brain. MBTs tend to distribute along grey white junctions and watershed vascular distributions where the circulating tumor cells lodge in capillary beds prior to growing into symptomatic lesions [4]. MBTs are a major cause of morbidity and mortality in patients with metastatic cancer. The clinical symptoms of MBTs may be non-specific such as headaches from raised intracranial pressure or cognitive impairment or more specifically correlated to location and present with seizure or focal neurological deficits [5].

The brain, enclosed by the meninges, has a unique perivascular environment thanks to the blood-brain barrier (BBB), a selectively permeable tissue found around most blood vessels that limits the movement of molecules from the blood based upon molecular size and charge. The BBB prevents the entry of most hydrophilic chemotherapeutics, thereby acting as a refuge for metastatic tumors [6]. Furthermore, the BBB acts as an immune refuge, limiting exposure of the parenchyma to circulating antigens.

In addition, the BBB provides a microenvironment that is tightly metabolically regulated for the metastatic cells that have arrested in brain vasculature, extravasated, and begun to proliferate either in the perivascular space or the brain parenchyma. The brain’s interstitial fluid has high chloride content, enabling clones of neuroepithelial origin such as small cell carcinoma of the lung or melanoma to proliferate while sometimes inhibiting the growth of other cancer cell types lacking this predilection [7].

This review will focus on metastatic brain tumors, the hallmarks of the metastatic cascade, the genetics, and pathobiology of brain metastases. By understanding the molecular events necessary to sustain brain metastases, a foundation may be created to allow the investigation and development of more effective targeted therapies and research directions.

## 2. Pathogenesis of Brain Metastasis

To form a metastasis, a tumor cell must complete a sequential series of steps that begins with its detachment from the primary mass and invasion of the surrounding tissue.

The cascade involves two stages: (1) migration which includes intravasation, dissemination, and extravasation; (2) colonization.

### 2.1. Cellular Heterogeneity and Proliferation

The tumor cells are genetically heterogeneous, and their potential to metastasize is variable. Tumor cells can evade normal tissue organization, survive despite local environmental stresses such as hypoxia, nutrient deficiency, hypoperfusion, immune mediation, and can metastasize to distant sites. In the metastatic process, the cells are able to invade adjacent tissues, disseminate, adhere to new tissue substrates, and initiate neoangiogenesis. Tumor cells have the capacity to evade growth suppressors and inhibitors of cell proliferation via mechanisms including the resistance of apoptosis by overexpression of Bcl-2, Bcl-xL and down-regulation of pro-apoptotic Bax and Bim [8].

In normal tissue, epithelial cells are held in tight apposition by proteins serving the maintenance of structural integrity. Down-regulation of one of these proteins, *E-cadherins*, is correlated with high metastatic potential. Invading tumor cells secrete proteolytic enzymes that degrade the epithelial basement membrane. Migrating tumor cells then encounter the endothelial basement membrane of thin-walled blood vessels, which they must penetrate to enter the circulation. Tumor cells that arrest in capillary beds and gain access to the underlying tissue parenchyma activate angiogenic programs. These cells recruit adjacent microvascular endothelial cells as well as bone marrow derived circulating endothelial precursor cells to develop a new vascular supply [9] and increase the probability of further tumor cell dissemination.

In the primary tumors, there are heterogeneous cell lines ranging from cancer stem cells (CSC), partially differentiated progenitor cells, and fully differentiated end-stage cells which behave in an uncontrolled manner compared to normal tissue [10]. These CSCs may contribute to the enhanced malignant potential of primary tumors and are able to degrade the extracellular matrix, invade blood vessels and lymph nodes, migrate, extravasate, and colonize at their new locations [11,12].

### 2.2. Epithelial-Mesenchymal Transition (EMT)

Epithelial-Mesenchymal Transition (EMT) is a temporary, reversible phenomenon where cells can de-differentiate, migrate to a distant focus, and then re-differentiate to the original cell, forming a new structure [13]. Signals activating EMT are intrinsic (gene mutations) or extrinsic (growth factor signaling). Trans-differentiation is initiated by release of EMT inducing transcription factors that convert epithelial cells into mesenchymal derivatives, giving these cells the capacity to invade, resist apoptosis, and disseminate [14,15]. EMT enables non-CSCs to resemble a CSC state allowing them to invade and disseminate from the primary tumor to a distant, metastatic focus [16].

### 2.3. Interaction with Tumor Stroma

Cancer progression involves activation of cells in the adjacent stroma via paracrine signaling [17]. They include endothelial cells, pericytes, fibroblasts, and leukocytes with pro-tumorigenic factors to sustain tumor growth. The most prominent cells are the cancer-associated fibroblasts (CAF) and the pericytes. CAFs express high amounts of transforming growth factor beta (TGFβ), hepatocyte growth factor (HGF), epidermal growth factor (EGF), fibroblast growth factor (FGF), and IL-6 [18]. Experimental models suggest that cancer cells release factors such as CSF-1, which stimulate macrophages in the tumor microenvironment and release EGF promoting tumor proliferation [19]. The dynamic stromal environment stresses the tumor cells, enhancing genomic instability and epigenetic dysregulation [20].

### 2.4. Local Invasion

Once the phenotypically aggressive clone has developed, the tumor is spread via invasion of the extracellular matrix (ECM) with penetration into the vasculature and hematogenous dissemination to the CNS. Combined with the various molecular and cellular events, this leads to eventual tumor metastasis. Tumor cells must penetrate the vascular basement membrane to enter the circulation. The process is dependent on a number of protein complexes that regulate cellular interactions and proteolytic enzymes with degradation of the ECM, which permits extravasation.

### 2.5. E-Cadherin-Catenin Complex and Integrins

The E-cadherin-catenin molecular complex (ECCC) is essential for normal and tumoral cytoarchitecture. It is a mediator of cell-cell adhesion that determines cell polarity and organization [21]. Cadherin molecules are cell membrane glycoproteins that interact with each other in a homophilic manner with a stable extracellular fragment and a cytoplasmic protein coat, catenin. In tumor metastasis, tumor clones fail to adhere to one another, become disorganized, and are able to separate from the tumor mass. Infiltrating malignancies have mutations in the genes for catenins and *E-cadherins* which correlate with unfavorable prognosis. DNA hyper-methylation of *E-cadherin*’s promoter can diminish or silence its expression, disturbing the ECCC function, a common event in many metastatic cancers [22]. *N-cadherin* is another molecule connected to the cytoskeleton via α-catenin and β-catenin similar to *E-cadherin*. Gain-of-function mutations in *N-cadherin* also trigger increased migration and invasion in tumors [23].

Integrins are major adhesion and signaling receptor proteins linking the ECM to the cellular cytoskeleton and play an important role in mediating cell migration and invasion via signal transduction pathways, regulation of cytoskeletal organization, specific gene expression, control of growth, and apoptosis [24]. Integrins induce the release of a key signaling mediator, focal adhesion kinase (FAK). It is a ubiquitously expressed non-receptor cytoplasmic tyrosine kinase, thought to play a key role in migration and proliferation, by providing abnormal signals for survival, EMT, invasion, and angiogenesis [25].

The ability of tumor cells to escape the primary site is dependent on their ability to remodel the ECM by degrading the ECM via proteolytic enzymes, creating a pathway for invasion. Tumor cells carry this out by releasing signals that promote cell proliferation and angiogenesis in the metastatic cascade. Neurotrophins (NTs) promote brain invasion by enhancing the production of heparinase, an ECM proteolytic enzyme. Heparinase is a β-d-glucuronidase, s heparin sulfate degradative enzyme that destroys both the ECM and the BBB [26].

### 2.6. Dissemination

Once a tumor cell has breached its microenvironment and arrived at the vasculature or lymphatics, it must survive a stress environment. Tumor cells respond by reinforcing their cytoskeleton and adhering to the vascular wall [27]. By adhering to endothelium of target tissue, the tumor cells behave like macrophages, creating pseudopodia and penetrating the cell-cell junctions [28]. Cancer cells appear to attract platelets because of their expressed surface tissue proteins, which protect the cells from the immune system [29]. Once these mobile cancer cells get lodged in a secondary organ tissue site, they colonize by cellular diapedesis, extravasation and proliferation of the tumor cell mass, and by accumulation of tumor cells within the foreign tissue vascular bed; whereby, prior to rupture into adjacent stroma, they proliferate and begin to grow.

### 2.7. The Brain’s Microenvironment

The BBB is hypothesized to create and interact with a unique brain microenvironment and to influence metastatic colonization. The BBB consists of capillary endothelial cells that associate with tight junctions and that have no fenestrations. The endothelial cells are lined by pericytes, basement membrane, and astrocytes. There is low permeability to ions and small molecules and almost no permeability to macromolecules and peptides. There is no pinocytosis, a process which facilitates transcytosis. The BBB works with the blood-CSF barrier to protect the neural environment [30].

When tumor cells invade the BBB to establish a brain metastasis, endothelial cells form a blood-tumor barrier. Brain metastases are often associated with edema into the white matter surrounding the tumor, an effect influenced by the increased permeability of tumor-associated endothelial cells that permits leakage of proteins and water into brain parenchyma surrounding the tumor [31]. Glial cells provide structural support for neurons and influence brain and BBB integrity.

Microglia and macrophages secrete multiple cytokines, growth factors, enzymes, and reactive oxygen species that can directly or indirectly lead to angiogenesis (e.g., vascular endothelial growth factor (VEGF), tumor proliferation (e.g., EGF), and invasion (e.g., metalloproteases) of metastatic cancer cells in the brain [32]. In particular, studies on metastatic breast cancer cells show that their protein expression profile indicate that brain metastatic cells use enhanced mitochondrial respiratory pathways for energy production and antioxidant defense mechanisms [33]. The proteins up-regulated in the brain metastatic cells show three major changes in the energy metabolism: enhanced glycolysis, increased beta oxidation of fatty acids and an elevated pentose phosphate pathway [33]. The cells’ metabolic changes reflect adaptation of the tumor cells to the brain microenvironment where a constant high energy demand is met mostly by glucose oxidation [34,35]. The redox state of brain metastatic breast cancer cells may provide a link between their energy metabolism and gene regulation [33]. This shows the significance of understanding the metabolism of metastatic cells and supports the idea of targeting tumor energy metabolism for breast cancer brain metastases therapy.

### 2.8. Contribution of Systemic Immune Cells to Metastasis Proliferation on the Brain

For metastatic cancers that have arrested in the brain vasculature, extravasated and begun to proliferate either in the perivascular space or the brain parenchyma, the BBB provides a microenvironment that is tightly metabolically regulated. These tumor cells are not free from immune surveillance, either from CD95 (APO-1/Fas) ligands on the end processes of astrocytes and microglia or by exposure to extravasating systemic immune cells. Inflammation has been identified as an enabling characteristic of cancer, and there is a need for tumors to avoid immune destruction both in primary and metastatic tumors. A better understanding of tumor cell interactions with immune system cells is necessary thanks to an increasing move towards immunotherapy in cancer patients.

The pathways, by which infiltrating systemic immune cells can control the proliferation of metastatic cells in the CNS, include the resident CNS macrophages and mast cells, neutrophils, natural killer cells, antigen presenting cells, T and B lymphocytes, and platelets. Many of the hypotheses relating to mechanisms of metastatic extravasation in the brain have arisen from observations of *in vitro* and *in vivo* models [36,37]. First, tumor cells may be able to use similar mechanisms of those used by systemic immune cells to adhere to proteins expressed by endothelial cells allowing them to cross the BBB into the perivascular space; Second, tumor cells adhere to systemic immune cells via receptor-ligand interactions and cross through the BBB with the “hijacked” cells [38]; Third, tumor cells, mechanically arrested in blood vessels, may be able to modify the endothelial cell wall via expression of matrix metalloproteases (MMPs), allowing extravasation into the perivascular space [39].

## 3. Evolution of Brain Metastasis Animal Models

Progress has been made in modeling human cancer in the mouse which can recapitulate the range of phenotypes seen in primary human cancers. To date, no model of spontaneous brain metastasis has been developed, although cell lines injected intravenously have established tumor foci in brain and other organs [40].

### 3.1. Current Brain Metastases Models

Dr. Massague’s group investigates organ specificity by using an *in vivo* mouse model to identify MDA-MB-231-derived breast cancer lines with organotropism for bone and lung [41]. On rare occasions, foci of tumors grew in the brain; and after rounds of systemic injection, they were able to isolate outgrowth of cells with increased propensity of brain metastasis.

Dr. Werb described a model system of injecting human breast cells into fat pads of mice with a focus on determining when the cells acquire the ability to disseminate, colonize an organ, and grow. This model enables studying the interaction between tumor and stroma, including the function of microglia and inflammatory cells recruited to the site of tumor cell growth [35,37,40,41,42].

Reports in the literature suggest that other cell lines may be capable of brain metastasis *in vivo*, mimicking the heterogeneity in human cancer. They include a human cell line derived from a brain metastasis (MDA-MB-361), commonly studied lines such as MDA-MB-468, and rarely cited lines such as MA11 [30]. The carotid artery injections of MDA-MB-231 BR1 to BR3 sublines demonstrate that models can be robust enough to provide quantitation of therapeutic effects of compounds that may be used not only in molecular biology but also for preclinical drug development [30].

Traditionally, *in vitro* assays have been used to investigate motility, invasion of extracellular matrix, and anchorage-independent colonization. Dr. Lee’s laboratory investigated the invasion of human brain microvascular endothelial cells as a model for invasion of the blood brain barrier [43]. Invasion of labeled MDA-MB-231 cells could be measured quickly by assessing *in vitro* attachment to human brain microvascular endothelial cells and alterations in BBB properties.

Recently, the authors have been working on creating both patient-derived *in vivo* and *in vitro* models of brain metastases originating from lung or breast cancer using surgical specimens, which will help identify therapeutically targetable genes.

### 3.2. Genetics in Breast Cancer Brain Metastases

There is an urgent need to better understand the mechanisms underlying the pathogenesis of brain metastasis and to identify novel targeted therapies. Gene expression studies using DNA microarrays have identified at least four distinct subtypes of breast cancer, including Luminal A, Luminal B, HER2+/ER−, and basal-like subtypes [44,45]. Dr. Salhia’s research focuses on comprehensive genetics and epigenetics analysis with microarray technology to measure alterations at the level of mRNA expression, DNA copy number, and DNA methylation.

A number of regions of amplifications and deletions have been identified using copy number analysis. The most notable regions of broad gains were 1q, 5p, 8q, 11q, and 20q. Broad deletions were identified at 8p, 17p, 21p, and Xq. Studies have shown that invasive breast cancer has shown deletions in 1p, 8p, 11q, 16q, 18q, and 22 [46,47]. This suggests overlap of regions involved in primary breast cancer, pointing towards emergence of chromosomal abnormalities unique to breast cancer metastasis. Breast cancer oncogene such as Myc (8q) were not highly expressed in Dr. Salhia’s lab [48]. However, ATAD2 (8q24) and DERL1 (8q24) were overexpressed and amplified suggesting a role in breast cancer metastasis. ATAD2 is a transcriptional coactivator of ESR1 required to induce estradiol target gene expression [49]. DERL1 is thought to participate in the endoplasmic-reticulum (ER) associated degradation response and proteosomal degradation.

Differential expression analysis revealed significant profiles associated with G2-M checkpoint and proliferation. FOXM1, a transcriptional activator in the G2-M cascade, was overexpressed in a large percentage of breast cancer metastases. FOXM1 regulated many genes involved in the mitotic checkpoint such as AURKA, AURKB, PLK1, CENPF.

## 4. Epigenetics in Brain Metastases

### 4.1. DNA Methylation

Cancer-associated localized hypermethylation has been described for CpG islands, DNA sequences of about 1–2 kb that are (C+G)-rich. When their promoters overlap, CpG islands are usually unmethylated in tissues. Frequent and excessive hypermethylation of CpG islands is observed in many cancers. However, DNA hypomethylation in cancer often affects more of the genome than does hypermethylation so that net losses of genomic 5-methylcytosine are seen in many human cancers [50]. It is important to consider DNA hypomethylation to tumorigenesis in light of cancer therapies involving decreasing DNA methylation. Inducing DNA-hypomethylation may have short term effects, but may also help speed tumor progression from cancer cells surviving the DNA methylation chemotherapy [51]. A recent study in Dr. Salhia’s lab showed increased DNA methylation levels compared to non-neoplastic tissue, which is likely due to amplification and overexpression of DNMT3B and MAT1A. Compared to HER2 enriched and luminal B brain metastasis, there was overall decreased methylation in basal-like tumors, consistent with primary breast cancer findings (TCGA) [52].

### 4.2. MicroRNAs in Brain Metastases

Research on the metastatic cascade has demonstrated the role of incorrectly regulated protein expression. Molecular studies have emphasized the role of microRNAs. They are a large class of small non-coding RNAs, 19–25 nucleotides long, and are produced naturally in cells after being cut into segments from larger strands of RNA. They bind to complementary sites on the 3'UTR of genes and promote the recruitment of protein complexes responsible for impairing translation and/or decreasing the stability of mRNA [53]. MicroRNAs in cancer cells are atypically expressed compared to normal cells. MicroRNA profiles have been shown to distinguish between primary and secondary brain tumors and categorize metastatic brain tumor of origin [2]. This suggests that microRNA profiling may be used to identify cancer cell origin and its response to therapy.

**Figure 1:**
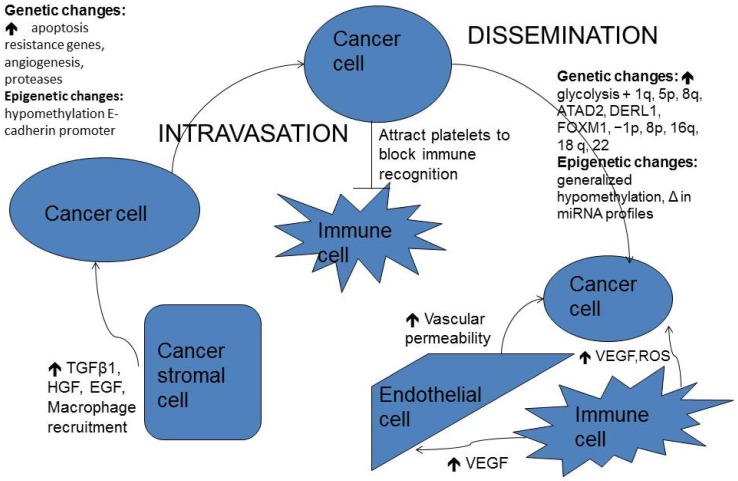
Overview of cancer cell interactions with stromal and immune cells, genetic and epigenetic changes during the metastatic cascade.

## 5. Conclusions

The metastatic cascade is a complex, multi-step process (Figure 1). Involvement of the CNS in patients with metastatic disease is increasingly more treatable. Brain metastases are becoming increasingly more prevalent as greater control over systemic disease is achieved and large molecules such as antibodies are used in the control of primary cancers. Due both to the BBB and the brain’s unique microenvironment, novel approaches for the treatment of brain metastases will be necessary.

Recent advances in animal models of experimental metastases to the brain will facilitate molecular analyses and the development of pre-clinical studies. It is possible that increased specificity in targeting will lead to increased efficacy and reduced toxicity in the clinical care of those with MBTs. A more thorough understanding of the metastatic process will enable more precise therapeutic targeting of metastases and may lead to earlier detection and improved treatment of brain metastases.
